# FXR agonist in post-liver transplantation patients: a randomized open-labeled study.

**DOI:** 10.12688/f1000research.159202.2

**Published:** 2025-06-27

**Authors:** Anila K N, Saraswathy S Nair, Dinesh Balakrishnan`, Unnikrishnan G, Binoj S T, Ramachandran Narayana Menon, Christi Titus Varghese, Shweta Mallick, Krishnanunni Nair, Madhu Srinivasan Durairaj, Guhan V, Nafiya Zackariah, Haritha Rajakrishnan, Arun Valsan, Johns Shaji Mathew, Cyriac Abby Philips, Christopher Watson, Sudheer O V, Sudhindran S

**Affiliations:** 1 GI Surgery, Amrita Institute of Medical Sciences & Research Centre, Kochi, Amrita Vishwa Vidyapeetham, Coimbatore, Tamil Nadu, India; 2 Hepatology, Division of Gastromedicine, Amrita Institute of Medical Sciences & Research Centre, Amrita Vishwa Vidyapeetham, Coimbatore, Tamil Nadu, India; 3 Department of Multiorgan transplant and HPB Surgery, Burjeel Hospital, Abu Dhabi, Abu Dhabi, United Arab Emirates; 4 Department of Clinical and Translational Hepatology, The Liver Institute, Rajagiri Hospital, Aluva, Kerala, India; 5 Addenbrookes Hospital, Cambridge University Hospitals NHS Foundation Trust, Cambridge, CB2 OQQ, UK

**Keywords:** Liver transplantation, cholestasis, ursodeoxycholic acid, obeticholic acid, alkaline phosphatase.

## Abstract

Liver diseases cause nearly 2 million deaths worldwide each year, with approximately 1 million deaths from cirrhosis complications and another 1 million from viral hepatitis and liver cancer, according to WHO estimates. Liver transplantation (LT) remains the primary curative option, boasting success rates of 85% in the first year and 75% at five years post-transplant. Despite high costs, LT is considered cost-effective, especially for younger patients with active work years remaining. However, post-transplant complications, particularly intrahepatic cholestasis, present notable challenges. This complication arises from factors such as ischemia-reperfusion injury, infections, immunological rejection, and surgical complications, all contributing to impaired bile flow and liver damage. Current medical therapies for post-LT cholestasis are limited, with ursodeoxycholic acid (UDCA) frequently used, despite questionable efficacy. Obeticholic acid (OCA), a potent Farnesoid X Receptor (FXR) agonist approved by the FDA for treating primary biliary cholangitis (PBC), has shown potential benefits in reducing elevated cholestatic liver enzymes. Given its significant effects on liver health, OCA may offer therapeutic value in managing post-transplant cholestasis and improving graft survival.

This randomized controlled trial (RCT) aims to evaluate OCA’s efficacy compared to UDCA in reducing cholestatic injury and enhancing graft function post-LT. The primary outcomes will focus on a 15% reduction in alkaline phosphatase and gamma-glutamyl transferase levels at 3, 6, and 12 months from baseline one month. Secondary outcomes include molecular markers, biliary complications, graft rejection, quality of life, and cost-effectiveness. Results are anticipated to demonstrate that OCA could improve graft survival, reduce complications, and enhance quality of life, potentially setting a new standard in post-LT care if found beneficial.

## Introduction

Liver diseases cause nearly 2 million deaths worldwide each year, with approximately 1 million deaths from cirrhosis complications and another 1 million from viral hepatitis and liver cancer, according to WHO estimates. ^1, 2^ Liver transplantation (LT) is recognized as the primary curative treatment, with success rates reaching 85% in the first year and 75% at 5 years post-transplantation. ^3^ In India, over 3000 liver transplants are performed annually. Despite the considerable expense associated with LT, it is considered cost-effective, especially for individuals in their prime years, who have many active work years ahead of them. ^4, 5^ However, post-transplant morbidity, particularly intrahepatic cholestasis, presents significant challenges. ^6^

Intrahepatic cholestasis is a major complication following liver transplantation, caused by multiple factors. Ischemia-reperfusion injury during organ retrieval and transplantation leads to cellular damage and postoperative cholestasis. ^7, 8^ Sepsis from infections worsen cholestasis through inflammatory responses. ^9^ Essential post-transplant drugs, including immunosuppressants and antibiotics, can interfere with bile formation, causing liver injury. ^10, 11^ Immunological reactions such as T-cell and B-cell-mediated rejection and lymphocytic cholangitis also contribute to cholestasis. ^12^ Surgical complications like stenosis or obstruction of the biliary and vascular anastomosis can also result in intra- and extrahepatic cholestasis. ^13– 15^

It is usually administered for three months post-transplant at a dose of 10-15 mg/kg thrice daily. In 2016, the FDA approved obeticholic acid (OCA), a strong selective Farnesoid X Receptor (FXR) agonist, to treat primary biliary cholangitis (PBC) in conjunction with UDCA. ^16, 17^ Research has demonstrated that over 12 months, OCA, either in combination with UDCA or on its own, dramatically lowers levels of total bilirubin and alkaline phosphatase. ^18, 19^ In individuals with advanced liver disease, the FDA has cautioned against administering OCA at doses of 10–50 mg. ^20^ Studies comparing OCA’s effects on cholestasis, rejection, regeneration, and cost-effectiveness to the current standard UDCA are being conducted to assess the role of OCA in the postoperative recovery of liver transplant patients. ^21^ Our proposed project is a clinical trial between OCA and the current standard of care, UDCA, in managing post-liver transplant cholestasis and improving graft survival. ^22^

### Rationale

Following LT, a major morbidity mitigating long-term liver functions is intrahepatic cholestasis due to a multitude of reasons. The only drug available for the treatment of intrahepatic cholestasis currently is UDCA. ^23^ FXR is a newly detected receptor gene, playing multiple roles in liver homeostasis, including detoxification of bile acid excess, stimulation of bile salt export from hepatocytes, reduction of inflammation, delaying fibrosis, and perhaps may even assist liver regeneration. ^24, 25^ A promising alternative is obeticholic acid, that has got its approval by the FDA for cholestatic conditions like primary biliary cholangitis (PBC). ^26^ Research shows OCA significantly reduces key liver enzymes like alkaline phosphatase, gamma glutamyl transferase and total bilirubin in cholestatic liver disease, though its effects specifically in LT patients have not been well-studied. ^27, 28^

This study aims to fill an essential gap by evaluating the efficacy of obeticholic acid compared to ursodeoxycholic acid in managing post-liver transplant cholestasis. By conducting specific biochemical tests to assess cholestasis, this research will provide insights into the effectiveness of OCA relative to the current standard, UDCA. ^29^ Additionally, this study will examine the potential effects of OCA on rejection, tissue regeneration, and cost-effectiveness—areas where knowledge is currently limited. ^30, 31^ The ultimate goal of this study is to determine whether OCA can emerge as a new standard of care in post-LT management, offering cost-effective solutions to enhance long-term outcomes for liver transplant patients.

### Primary outcomes

We are assessing the effects of obeticholic acid compared to ursodeoxycholic acid in post-liver transplant patients in ameliorating cholestatic injury with primary endpoints, a 15% reduction in alkaline phosphatase and gamma-glutamyl transferase levels at 3, 6, and 12 months from one-month baseline between groups.

### Secondary outcomes

• Assessment of biochemical markers (Liver function tests, metabolic profile, CRP, tacrolimus levels).
• Molecular markers (fibroblast growth factor 19 (FGF-19), transforming growth factor β (TGF-β) level, Cytokeratin 18 level, Serum autotaxin level, Bile Salt Export Pump levels (BSEP), Plasma bile acid levels.
• Biopsy-proven acute or chronic rejection.
• Incidence of biliary complications.
• All-cause mortality.
• Any adverse events.
• Death due to acute or chronic rejection.
• Quality of life.

## Methods

This RCT is being conducted in the Department of GI Surgery, Amrita Institute of Medical Sciences, Kochi.

### Study design and participants

The study is being conducted as an investigator-initiated, parallel-group, open-label randomized controlled trial in patients undergoing live donor liver transplantation since December 2021 in the Department of Gastrointestinal Surgery at a tertiary care teaching hospital.

### Eligibility & exclusion criteria

All adult patients undergoing live donor liver transplantation during the study period are being included. Recipients who are less than 18 years of age, those undergoing deceased donor liver transplants, ABO-incompatible transplants, retransplant cases, those not surviving beyond 30 days, and patients lost to follow-up during the study period, are being excluded. We excluded patients who were randomized into the trial but died within the first 30 days. This decision was based on the fact that early mortality in this period is most commonly attributable to sepsis or technical complications. Including such patients would not be appropriate for evaluating interventions aimed at the management of cholestasis, which usually occur 2 to 3 weeks after surgery. Mortality within 30 days, purely secondary to the adverse effect of the drug, with no other technical (vasculo-biliary), septic or immunological cause is extremely unlikely.

### Recruitment & randomization

A clinical pharmacist is randomizing study participants admitted to the GI Surgery ICU following liver transplantation. A permuted block sequence of computer-generated random numbers is being used to enroll subjects into either the control arm (C) or the study arm (T)—i.e., to receive either the standard liver protectant ursodeoxycholic acid [URSETOR ^®^ of Torrent Pharma] 10–15 mg/kg TID (control) or obeticholic acid 5 mg OD [OCANASH ^®^ of Macleods Pharmaceutical Limited]. The sequence is being kept in sealed opaque envelopes and is being opened by the pharmacist on the second day following liver transplantation.

Given the difference in tablet size and dosing frequency (three times daily for UDCA vs once daily for OCA), blinding is not being done.

**Figure 1.**
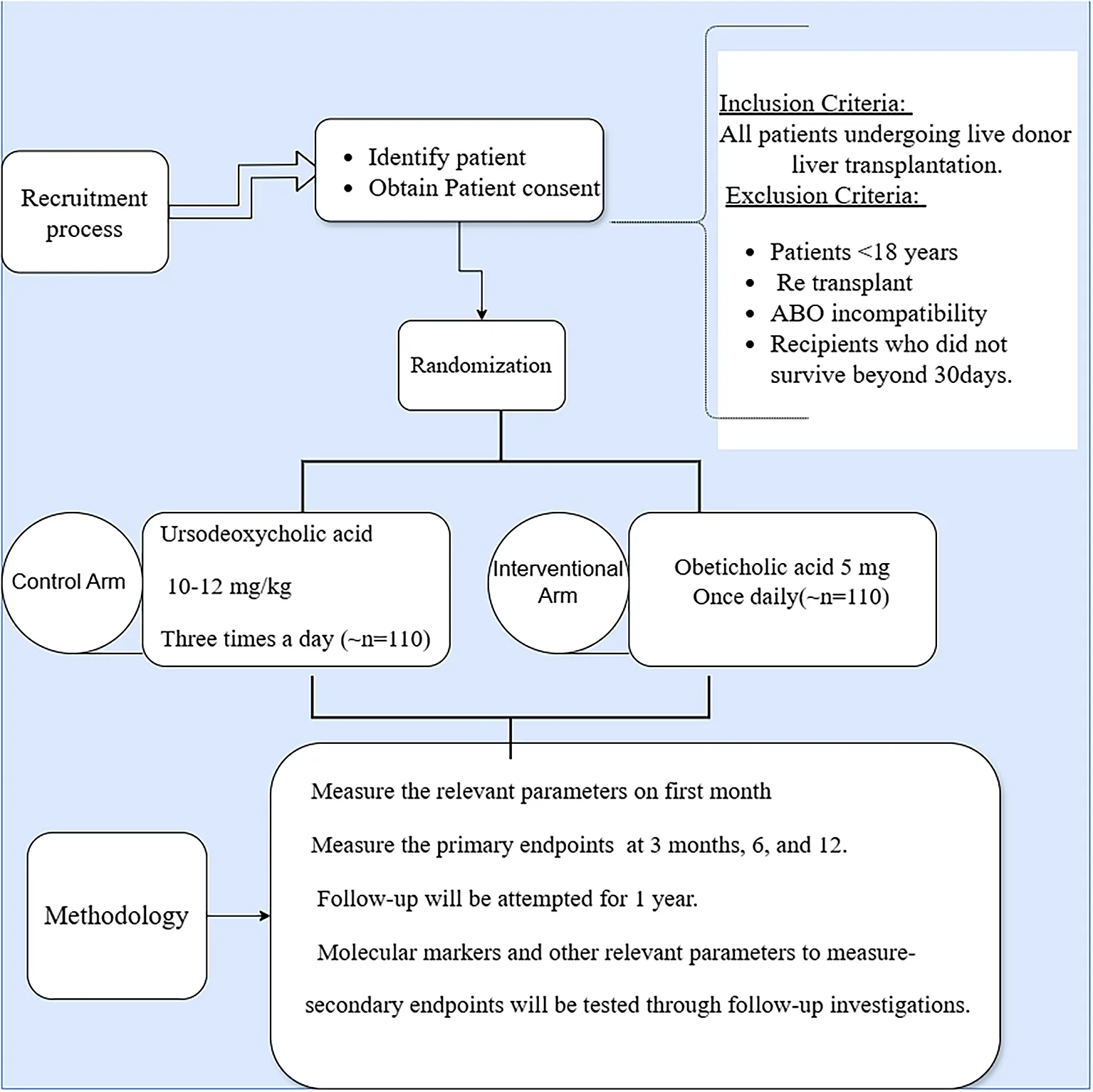
Flow chart showing study methodology.

Follow-up visits for physical examinations and safety assessments are being scheduled during the study period at the following intervals: one month, three months, six months, and twelve months post-transplant ( Figure 1). Liver function tests, C-reactive protein (CRP), platelet count, prothrombin time/international normalized ratio (PT/INR), fasting blood sugar (FBS), tacrolimus concentration, and other relevant parameters are being measured at each follow-up visit. Lipid profile—including total cholesterol, low-density lipoprotein (LDL), high-density lipoprotein (HDL), very low-density lipoprotein (VLDL), and triglycerides (TG)—as well as glycosylated hemoglobin (HbA1c) levels, are being assessed at pre-transplant admission, and at three, six, and twelve months post-transplant.

We selected a 15% reduction in both alkaline phosphatase (ALP) and gamma-glutamyl transferase (GGT) as our primary biochemical endpoint based on evidence from previous trials in cholestatic liver disease. Specifically, the landmark study by Nevens et al., published in The New England Journal of Medicine (2016), evaluated obeticholic acid in patients with primary biliary cholangitis and used a composite endpoint that included a ≥15% reduction in ALP as part of the therapeutic response criteria.

Although this trial was not conducted in post–liver transplant patients, it provided a strong precedent for using ALP-based endpoints to assess treatment response in cholestatic conditions. We extended this rationale by including GGT alongside ALP, as the combination of these two enzymes provides a more comprehensive and sensitive assessment of cholestasis, particularly in the post-transplant setting where bile duct injury and graft function are dynamic.

Given the lack of clinical trials of OCA in the liver transplant population, this combined biochemical endpoint serves as a reasonable and evidence-informed surrogate marker of response in our study.

Secondary endpoint serum markers are being assessed using an enzyme-linked immunosorbent assay (ELISA) [ELK Biotechnology™] at the centralized biochemistry laboratory. Blood samples collected during the one-month and twelfth-month post-transplant follow-up visits are being used for ELISA testing.

In addition, demographics, patient characteristics, and other critical study details (e.g., medication history, medical history, social habits, family history, and use of other medical systems) are being collected through direct patient or bystander interviews, doctor consultation notes, hospital health information systems, and case records. The collected data is being transcribed into a pre-designed proforma (data collection form) by the investigator.

### Biopsy and rejection evaluation

A biopsy is being performed when suspected rejection (unexplained rise in transaminases) is observed. The biopsy results are being evaluated based on the Rejection Activity Index score and the Banff criteria. Data on the frequency and duration of the first biopsy-proven acute rejection (BPAR) episode (within six months of transplantation) that requires therapy are being recorded. Any biliary system-related strictures, leaks, or anastomosis issues are being documented. Early allograft dysfunction (EAD) is being assessed using the criteria developed by Olthoff et al. ^7, 32^

### Adverse reactions and causality assessment

Adverse events are being captured by direct interview of patients during the routine follow-up visits. We are capturing the pruritus by physical examination, vital signs and 5D pruritus questionnaire and documented systematically using structured Case Report Forms (CRFs) to ensure standardized data collection and analysis. The Naranjo algorithm will be used to establish causality and then classify them as definite, probable, possible, or unlikely.

### Quality of life (QOL) measurement

During patient interviews, a validated Chronic Liver Disease Questionnaire (CLDQ) is being administered to assess quality of life (QOL). ^33^ The copyright license for the English version of the questionnaire is being obtained from the authors. The QOL questionnaire is being completed both pre-transplant and at the one-year post-transplant follow-up. Patients are completing the questionnaire in either Malayalam or English.

The CLDQ includes six domains:

• **Abdominal symptoms (AB)**: Items 1, 5, 17
• **Fatigue (FA)**: Items 2, 4, 8, 11, 13
• **Systemic symptoms (SY)**: Items 3, 6, 21, 23, 27
• **Activity (AC)**: Items 7, 9, 14
• **Emotional functions (EM)**: Items 10, 12, 15, 16, 19, 20, 24, 26
• **Worry (WO)**: Items 18, 22, 25

The CLDQ overall score is the sum of the scores from each domain, ranging from 0 to 203. Lower scores indicate poorer quality of life.

### Sample size

The sample size was calculated based on a pilot study comparing the effect of obeticholic acid (OCA) and ursodeoxycholic acid (UDCA) on cholestatic injury in post-liver transplant patients. In the pilot study, the difference in alkaline phosphatase (5.131 ± 39.639 vs 12.496 ± 52.139) was observed between the two groups. With 80% power and 95% confidence, the minimum sample size required was determined to be 108 patients in each group, totaling 216 samples. We will be recruiting around 110 to 120 patients per arm.

### Study status

The study is currently in the recruitment phase.

### Statistical analysis

• To compare the means of continuous variables between groups (Drug A vs Drug B), an independent sample t-test will be used at each time point.
• To compare the changes in continuous variables from pre- (1 month) to post-transplant (3, 6, and 12 months), a paired t-test will be applied.
• To compare the means of variables from baseline to subsequent time points within each group, repeated measures ANOVA will be used if significant. The Bonferroni multiple comparison test will be applied for pairwise comparisons.

## Expected results

We expect a significant reduction in cholestatic liver enzymes following LDLT in the OCA group compared to the UDCA group, with improvements observed from one month to subsequent time points (3, 6, and 12 months). We hypothesize that the potent mechanism of action of OCA will ameliorate cholestatic injury and enhance graft survival. Additionally, we anticipate a reduction in biliary complications, all-cause mortality, and an increase in quality of life for OCA-receiving patients. This may reduce long-term financial expenditure for patients. Given that there are currently no drugs to prevent damage to the transplanted liver, a positive outcome could be a significant advancement for the transplant population. If beneficial, this study could lead to the adoption of OCA as a standard of care following liver transplantation.

## Conclusion

Obeticholic acid (OCA) is a farnesoid X receptor (FXR) agonist, with significantly more potency than UDCA in modulating bile acid metabolism. While OCA has shown promise in treating cholestatic liver diseases, its effects post-liver transplantation (LT) remain unclear due to the lack of high-quality trials. Our study aims to evaluate whether OCA’s strong FXR activation can benefit patients with post-LT cholestasis. We hypothesize that OCA will improve graft function, reduce biliary complications, lower associated costs, and enhance overall quality of life and survival for post-LT patients.

### Confidentiality

All information about participants enrolled in this clinical trial are treated with the utmost confidentiality to protect their privacy and adhere to ethical standards. Personal identifying information are securely stored and accessible only to authorized personnel involved in the study. Data collected are anonymized and coded to maintain confidentiality during analysis and reporting. Any publication or presentation of results will not include identifiable information unless explicit consent is obtained from participants.

### Access to data

Access to data collected during this clinical trial will be managed according to established protocols and regulatory requirements. Authorized personnel, including investigators, statisticians, and regulatory authorities, will have access to raw data for analysis, monitoring, and verification. Data-sharing agreements will be established to govern access by external parties, ensuring compliance with data protection regulations and participant confidentiality. Requests for access to study data from external researchers or institutions will be considered on a case-by-case basis and subject to review by the study sponsor and relevant ethics committees.

### Data dissemination

Upon completion of the study, data will be disseminated through peer-reviewed journal publications, conference presentations, and participant communication. Findings will be shared transparently with participants, addressing risks and benefits. Regulatory reporting will ensure compliance with obligations to relevant agencies. Additionally, data-sharing agreements and repositories will facilitate access for other researchers while maintaining participant confidentiality.

### Ethical considerations

The study protocol was initially approved by the Institutional Ethics Committee, AIMS (IEC-AIMS-2021-GISURG-262) on 13-10-2021 and later modified for a title change on 15-06-2022 (modified ethics number—IEC-AIMS-2022-GISURG-160). This study involves liver transplant patients aged 18 years or older admitted to our hospital’s gastrointestinal surgery department. Before the study’s initiation, patients gave written informed consent. The consent form was prepared both in English and the local language Malayalam. The study has received ethical clearance from our Institutional Ethics Committee. The committee also approved the informed consent in both languages. This study was registered with the Clinical Trials Registry of India. Our CTRI registration number is CTRI/2021/11/038218 dated on 23/11/2021. The study was conducted according to the principles of the Declaration of Helsinki.

## Author contributions

• S. Sudhindran, Unnikrishnan G, Dinesh Balakrishnan, Johns Shaji Mathew, and Shweta Mallick contributed to formulating the concepts.
• Implementation of study including randomization, and data collection: Anila KN, Saraswathy S Nair, Nafiya Zackariah, Haritha Rajakrishnan.
• Drafting manuscript: Anila K N.
• S. Sudhindran, OV Sudheer, Arun Valsan, Binoj ST, Ramachandran Narayana Menon, Krishnanunni Nair, Madhu Srinivasan Durairaj, V Guhan, Christi Titus Varghese contributed to giving valuable inputs and critical revisions.

## Data Availability

No data are associated with this article. Open Science Frame (OSF): FXR agonist in post Liver transplantation patients: A randomized open-labeled study, DOI: 10.17605/OSF.IO/JDXBF ^34^ This project contains the following underlying data:
1. CLD malayalam questionnaire. Pdf 2. CLD QUESTIONNAIRE english.pdf 3. Data collection CRF.pdf 4. Ethics docs.zip 5. Fillable-SPIRIT-Outcomes-2022-Checklist-with-SPIRIT-2013.pdf 6. INFORMED CONSENT DOCUMENT English final.pdf 7. Informed consent Malayalam modified.pdf Data are available under the terms of the Creative Commons Zero “No Rights Reserved” data waiver (CC0 1.0 Public Domain Dedication).
