# Supplementary material for: FXR agonist in post-liver transplantation patients: a randomized open-labeled study. [Protocol]
Source: F1000Res. 2026 Mar 11;14:9. Originally published 2025 Jan 2. [Version 3] doi: 10.12688/f1000research.159202.3 (PMC13560005; doi:10.12688/f1000research.159202.3)
Supplement: Peer review history

# Reviewer report: Reviewer response for version 3

**DOI:** 10.5256/f1000research.196925.r488192

**Published:** 2026-08-11

**Authors:** Sonal Sekhar

**Affiliations:** 1 Manipal Academy of Higher Education, Manipal, Karnataka, India

This protocol addresses a vital and clinically relevant gap in managing post-liver transplant cholestasis. The design of the study (RCT) is appropriate, and the revised version has improved significantly based on prior reviewer comments. 

 Strength: The rationale for evaluating FXR agonists (OCA) in post-LT cholestasis is strong and biologically plausible.

 The manuscript clearly identifies the unmet need, as UDCA has limited efficacy.

 However, few methodological, and reporting clarifications are needed to enhance scientific rigor and reproducibility.

 1. Lack of blinding introduces performance and detection biases, even if biochemical outcomes are objective.

 2. Exclusion of patients dying within 30 days is justified based on early postoperative confounders.

- This may introduce survivor bias, excluding patients with severe events.
- It potentially limits generalizability and safety interpretation.

Is the study design appropriate for the research question?

Yes

Is the rationale for, and objectives of, the study clearly described?

Yes

Are sufficient details of the methods provided to allow replication by others?

Yes

Are the datasets clearly presented in a useable and accessible format?

Yes

Reviewer Expertise:

Diabetes, Tuberculosis, Pharmacoepidemiology

I confirm that I have read this submission and believe that I have an appropriate level of expertise to confirm that it is of an acceptable scientific standard.

# Reviewer report: Reviewer response for version 1

**DOI:** 10.5256/f1000research.174896.r377804

**Published:** 2025-05-15

**Authors:** Gregory Guthrie

**Affiliations:** 1 Baylor College of Medicine, Houston, Texas, USA

This manuscript addresses an important clinical question: the optimal management of post-liver transplant (LT) cholestasis, comparing Obeticholic Acid (OCA) with the current standard, Ursodeoxycholic Acid (UDCA). The rationale for exploring FXR agonists like OCA in this setting is sound, given their known roles in bile acid homeostasis, inflammation, and potentially regeneration.

 However, for the manuscript to be considered for indexed, several weaknesses need to be addressed, and significant clarifications and additions are required.

**Major Weaknesses and Areas Requiring Clarification/Addition:**

1. **Study Timeline and Design Clarity (Critical Flaw):**
  - **Contradiction:** The "Methods" section states the study "was conducted... from December 2014 to 2021." However, the "Study status" section says, "The study is currently in the recruitment phase." Furthermore, ethics approval is dated 2021/2022, and CTRI registration is from 2021. This is a fundamental contradiction. My understanding is that it is patients that received LT in these years that will be recruited to the study, but the constant change is present and past tense, and the wording makes this unclear. Furthermore, exclusion criteria for patients that died within 30 days would also suggest a difference in timeline, as a patient who died in 2014 after 30 days post LT would certainly not be available to include in a study taking place in 2025. Otherwise, is this exclusion criteria for patients that are started on the study and die within this time frame, in which case this could exclude patients experiencing severe early adverse events or graft failure potentially related to the interventions. A rationale for this exclusion is needed.
2. **Rationale for OCA Dosage and Safety in LT Population:**
  - **FDA Warning Context:** The introduction mentions, "In individuals with advanced liver disease, the FDA has cautioned against administering OCA at doses of 10–50 mg." Liver transplant recipients, by definition, have end-stage liver disease pre-transplant and a recovering, vulnerable graft post-transplant. While the study uses 5 mg OD, which is lower, the potential for OCA-related complications (decompensation, worsening LFTs before improvement, severe pruritus) in this specific population needs more thorough discussion.
3. **Primary Endpoint Justification and Baseline:**
  - **15% Reduction:** The primary endpoint is a "15% reduction in alkaline phosphatase and gamma-glutamyl transferase levels." What is the clinical significance of a 15% reduction in these enzymes in post-LT cholestasis? Is this threshold based on previous studies in LT or other cholestatic conditions demonstrating improved long-term outcomes? This needs robust justification.
  - **One-Month Baseline:** The baseline for assessing the primary endpoint is "one-month" post-transplant. Significant cholestasis and LFT fluctuations can occur much earlier post-LT. Why was a 1-month baseline chosen? This might miss early effects or be confounded by stabilization that might occur irrespective of the study drug. A rationale for this time point is crucial.
    - **Omission of Bilirubin:** Total bilirubin is a key marker of cholestasis severity and liver function. Why is bilirubin not included as a primary or at least a key secondary endpoint for cholestasis assessment?
4. **Sample Size Calculation and Pilot Study Data:**
  - The pilot study data used for sample size calculation ("difference in alkaline phosphatase (5.131 ± 39.639 vs 12.496 ± 52.139)") shows extremely large standard deviations relative to the means. This suggests massive variability or heterogeneity in the pilot data, or potentially a very small effect size, making the observed difference potentially unreliable for powering a larger trial. A difference of ~7 U/L (assuming these are absolute values) seems very small.
5. **Exclusion Criteria and Analysis Population:**
  - "patients who discontinued study drugs were excluded." Excluding patients who discontinue the drug from the analysis can introduce significant bias. An intention-to-treat (ITT) analysis, including all randomized patients in the groups to which they were originally assigned, is standard for RCTs and should be the primary analysis. A per-protocol analysis can be secondary. This needs to be specified in the statistical analysis section.
  - See earlier comment on exclusion criteria of patients that died <30 days.

**Secondary Weaknesses and Areas for Clarification/Addition:**

1. **UDCA Dosing:**
  -
    - UDCA is dosed at "10-15 mg/kg TID." Typically, UDCA is administered BID in post-transplant settings. Provide a rationale for the TID dosing regimen for UDCA.
2. **Measurement of Specific Secondary Endpoints:**
  -
    - "Bile Salt Export Pump levels (BSEP)": How will BSEP levels be measured? Perhaps I am mistaken, but BSEP is a membrane-bound transporter, so will only be measured with biopsies, as in you methods, will only be done in the suspicion of rejection. This is currently too vague.
  - "Plasma bile acid levels":
    - **Clarification Needed:** Will this be total plasma bile acids, or will specific bile acid species be fractionated and quantified (e.g., via LC-MS/MS)? The latter would be much more informative regarding FXR target engagement and changes in bile acid pool composition.
3. **Definition of "Cellular Level" Assessment:**
  - The Rationale mentions "specific biochemical tests to assess cholestasis at the cellular level."
    - **Clarification Needed:** Which specific tests (beyond standard LFTs and the listed secondary markers) are being referred to that assess cholestasis at a "cellular level"? This phrasing needs to be more precise or linked directly to specific planned assays (e.g., markers of oxidative stress, apoptosis, or specific transport protein expression if biopsies are analyzed beyond histology).
4. **Introduction and Conclusion Phrasing:**
  - Introduction: "Although its usefulness is questionable, many practitioners utilize ursodeoxycholic acid (UDCA)..." While there are debates, phrasing it as "questionable" without immediate specific referencing for the LT context might be too strong. Perhaps "its efficacy for preventing all forms of post-LT cholestasis or improving long-term graft survival has limitations" or similar.
  - Conclusion: "Obeticholic acid (OCA) is a potent farnesoid X receptor (FXR) agonist, with 100 times the efficacy of the current standard treatment, UDCA..." This is misleading. OCA is ~100 times more potent as an *FXR agonist* than chenodeoxycholic acid (a primary bile acid), not UDCA. UDCA is not a potent FXR agonist; it has different mechanisms of action (choleretic, cytoprotective, immunomodulatory). This statement needs correction for accuracy.

The study addresses a relevant clinical need. However, the current manuscript contains fundamental inconsistencies and lacks necessary detail and justification in several key areas. Addressing these points comprehensively will be essential for its scientific validity and potential for indexing.

Is the study design appropriate for the research question?

Yes

Is the rationale for, and objectives of, the study clearly described?

Yes

Are sufficient details of the methods provided to allow replication by others?

No

Are the datasets clearly presented in a useable and accessible format?

Partly

Reviewer Expertise:

My research is focused on mechanisms and treatment of cholestatic liver diseases.

I confirm that I have read this submission and believe that I have an appropriate level of expertise to confirm that it is of an acceptable scientific standard, however I have significant reservations, as outlined above.

# Response

**Published:** 2025-06-23

**Authors:** Anila K N

**Affiliations:** GI Surgery, Not Applicable, Kochi, Kerala, India

**Thank you very much for the very pertinent comments.**

**Major Weaknesses and Areas Requiring Clarification/Addition:**

**Query Study Timeline and Design Clarity (Critical Flaw):**

 Contradiction: The "Methods" section states the study "was conducted... from

 December 2014 to 2021." However, the "Study status" section says, "The study is

 currently in the recruitment phase." Furthermore, ethics approval is dated 2021/2022,

 and CTRI registration is from 2021. This is a fundamental contradiction. My

 understanding is that it is patients that received LT in these years that will be

 recruited to the study, but the constant change is present and past tense, and the

 wording makes this unclear.

**Reply:** Thank you for highlighting these points. We apologize for the mistake in the dates.

  The Institutional Ethics Committee approval for the study was obtained in August 2021, and the CTRI registration was obtained on 23/11/2021. The study commenced thereafter. The data collection period for the pilot study was mistakenly reported as December 2014 to 2021." and has now been corrected as given below. We have also addressed the inconsistencies in verb tense throughout the manuscript. We appreciate your careful review and constructive feedback.

 Correction in the manuscript:

**Study design and participants**

 The study is being conducted as an investigator-initiated, parallel-group, open-label randomized controlled trial in patients undergoing live donor liver transplantation since December 2021 in the Department of Gastrointestinal Surgery at a tertiary care teaching hospital.” under Study design and participants.

**Ethical considerations**

 The study protocol was initially approved by the Institutional Ethics Committee, AIMS (IEC-AIMS-2021-GISURG-262) on 13-10-2021 and later modified for a title change on 15-06-2022 (modified ethics number—IEC-AIMS-2022-GISURG-160). This study involves liver transplant patients aged 18 years or older admitted to our hospital’s gastrointestinal surgery department. Before the study’s initiation, patients gave written informed consent. The consent form was prepared both in English and the local language Malayalam. The study has received ethical clearance from our Institutional Ethics Committee. The committee also approved the informed consent in both languages. This study was registered with the Clinical Trials Registry of India. Our CTRI registration number is CTRI/2021/11/038218 dated on 23/11/2021. The study was conducted according to the principles of the Declaration of Helsinki.

**Query:**  Furthermore, exclusion criteria for patients that died within 30 days would also suggest a difference in timeline, as a patient who died in 2014 after 30 days post LT would certainly not be available to include in a study taking place in 2025. Otherwise, is this exclusion criteria for patients that are started on the study and die within this time frame, in which case this could exclude patients experiencing severe early adverse events or graft failure potentially related to the interventions. A rationale for this exclusion is needed.

**Explanation:** Thank you once again for pointing out these observations.

 The confusion is partly due to the mistake in the date, as the study started only in Dec 21. (which we have corrected as above)

  We excluded patients who were randomized into the trial but died within the first 30 days. This decision was based on the fact that early mortality in this period is most commonly attributable to sepsis or technical complications. Including such patients would not be appropriate for evaluating interventions aimed at the management of cholestasis, which usually occur 2 to 3 weeks after surgery. Mortality within 30 days, purely secondary to the adverse effect of the drug, with no other technical ( vasculo-biliary), septic or immunological cause is extremely unlikely. Furthermore, we require at least 4 to 6 weeks of drug therapy for any noticeable effect on cholestasis. Patients dying within 30 days will not get this time period.

 We have added this in the manuscript s follows: We excluded patients who were randomized into the trial but died within the first 30 days. This decision was based on the fact that early mortality in this period is most commonly attributable to sepsis or technical complications. Including such patients would not be appropriate for evaluating interventions aimed at the management of cholestasis, which usually occur 2 to 3 weeks after surgery. Mortality within 30 days, purely secondary to the adverse effect of the drug, with no other technical ( vasculo-biliary), septic or immunological cause is extremely unlikely.

**Query**: Rationale for OCA Dosage and Safety in LT Population:

  FDA Warning Context: The introduction mentions, "In individuals with advanced liver

 disease, the FDA has cautioned against administering OCA at doses of 10–50 mg."

 Liver transplant recipients, by definition, have end-stage liver disease pre-transplant

 and a recovering, vulnerable graft post-transplant. While the study uses 5 mg OD,

 which is lower, the potential for OCA-related complications (decompensation,

 worsening LFTs before improvement, severe pruritus) in this specific population

 needs more thorough discussion.

**Reply:** Thank you very much for your valuable comment.

 The FDA Blackbox warning was issued on the higher doses of obeticholic acid (10–50 mg) in individuals with advanced liver disease. As you correctly pointed out, liver transplant recipients represent a unique and vulnerable population with recovering graft function, and careful attention to OCA-related safety concerns is warranted.

 In our study, we have used a conservative dose of 5 mg once daily, which is well below the threshold associated with reported complications. Nevertheless, as this is an open-label trial, we are closely monitoring for potential adverse effects, including hepatic decompensation, transient worsening of liver function tests, and pruritus. These safety parameters are being actively assessed throughout the study duration and will be discussed comprehensively in the final analysis and manuscript.

**Query:** Primary Endpoint Justification and Baseline:

  15% Reduction: The primary endpoint is a "15% reduction in alkaline phosphatase

 and gamma-glutamyl transferase levels." What is the clinical significance of a 15%

 reduction in these enzymes in post-LT cholestasis? Is this threshold based on

 previous studies in LT or other cholestatic conditions demonstrating improved long

 term outcomes? This needs robust justification.

**Reply:**  We selected a 15% reduction in both alkaline phosphatase (ALP) and gamma-glutamyltransferase (GGT) as our primary biochemical endpoint based on evidence from previous trials in cholestatic liver disease. Specifically, the landmark study by Nevens et al., published in *The New England Journal of Medicine* (2016), evaluated obeticholic acid in patients with primary biliary cholangitis and used a composite endpoint that included a ≥15% reduction in ALP as part of the therapeutic response criteria.

 Although this trial was not conducted in post–liver transplant patients, it provided a strong precedent for using ALP-based endpoints to assess treatment response in cholestatic conditions. We extended this rationale by including GGT alongside ALP, as the combination of these two enzymes provides a more comprehensive and sensitive assessment of cholestasis, particularly in the post-transplant setting where bile duct injury and graft function are dynamic.

 Given the lack of clinical trials of OCA in the liver transplant population, this combined biochemical endpoint serves as a reasonable and evidence-informed surrogate marker of response in our study.

**Query:** One-Month Baseline: The baseline for assessing the primary endpoint is "one

 month" post-transplant. Significant cholestasis and LFT fluctuations can occur much

 earlier post-LT. Why was a 1-month baseline chosen? This might miss early effects or

 be confounded by stabilization that might occur irrespective of the study drug. A

 rationale for this time point is crucial.

**Reply:** We chose to initiate study assessments after one-month post-transplant because a significant proportion of our liver transplant recipients are high MELD patients who typically present with markedly elevated bilirubin and cholestatic enzyme levels at baseline. Additionally, in the immediate post-transplant period, transient elevations in liver function tests are commonly observed due to ischemia–reperfusion injury, surgical factors, sepsis, or early graft rejection.

 In live donor liver transplant for high MELD recipients, these fluctuations are often pronounced during the first few weeks following transplantation. Therefore, a 4-week window was provided to allow for stabilization of liver function parameters and resolution of early postoperative complications, ensuring that subsequent measurements more accurately reflect the effect of the study intervention rather than early perioperative variability.

 Query:  Omission of Bilirubin: Total bilirubin is a key marker of cholestasis severity

 and liver function. Why is bilirubin not included as a primary or at least a key

 secondary endpoint for cholestasis assessment?

**Reply:** Total bilirubin has been retained as an important secondary endpoint, given its clinical relevance in assessing hepatocellular function and persistent cholestasis. Indeed, liver function tests including bilirubin levels are listed as the first secondary end point.

**Query:** Sample Size Calculation and Pilot Study Data:

 The pilot study data used for sample size calculation ("difference in alkaline

 phosphatase (5.131 ± 39.639 vs 12.496 ± 52.139)") shows extremely large standard

 deviations relative to the means. This suggests massive variability or heterogeneity in

 the pilot data, or potentially a very small effect size, making the observed difference

 potentially unreliable for powering a larger trial. A difference of ~7 U/L (assuming

 these are absolute values) seems very small.

**Reply:** Regarding the sample size calculation, we acknowledge that data specific to the post–liver transplant population receiving obeticholic acid is extremely limited. Apart from our pilot study, we were unable to identify any previously published data in this specific setting to guide formal power calculations. Based on the effect sizes and variability observed in our pilot data, our statistician advised that this sample size would be adequate to provide preliminary evidence and inform the design of a larger, adequately powered trial in the future.

  Exclusion Criteria and Analysis Population:

  "patients who discontinued study drugs were excluded." Excluding patients who

 discontinue the drug from the analysis can introduce significant bias. An intention-to

 treat (ITT) analysis, including all randomized patients in the groups to which they

 were originally assigned, is standard for RCTs and should be the primary analysis. A

 per-protocol analysis can be secondary. This needs to be specified in the statistical

 analysis section.

 See earlier comment on exclusion criteria of patients that died <30 days.

**Reply: **Thank you very much for your observation. We are in fact conducting this trial as an intention-to-treat (ITT) analysis approach. Accordingly, all randomized patients—including those who discontinue the study prematurely—will be included in the final analysis. Efforts will be made to document and analyze the reasons for discontinuation, as this information is critical for understanding both the tolerability and the real-world applicability of the intervention **.**

**We have modified the exclusion criteria after removing this sentence as follows:**

 We excluded patients who were randomized into the trial but died within the first 30 days. This decision was based on the fact that early mortality in this period is most commonly attributable to sepsis or technical complications. Including such patients would not be appropriate for evaluating interventions aimed at the management of cholestasis, which usually occur 2 to 3 weeks after surgery. Mortality within 30 days, purely secondary to the adverse effect of the drug, with no other technical ( vasculo-biliary), septic or immunological cause is extremely unlikely.

 Query: Secondary Weaknesses and Areas for Clarification/Addition:

 UDCA Dosing:

 UDCA is dosed at "10-15 mg/kg TID." Typically, UDCA is administered BID in

 post-transplant settings. Provide a rationale for the TID dosing regimen for

 UDCA.

 Reply: Thank you for your comment regarding dosing. The typical recommended dosage for managing cholestasis—particularly with ursodeoxycholic acid (UDCA)—is **10 to 15 mg/kg/day**, usually administered in **three divided doses**. Based on this standard recommendation, we have selected the dose accordingly in our study to ensure therapeutic efficacy while maintaining safety and consistency with existing clinical guidelines.

 Several peer-reviewed studies and reviews that report the use of ursodeoxycholic acid (UDCA) at doses of 10–15 mg/kg/day in post–liver transplant patients for prophylaxis and early graft protection (Ref : Pedersen, Mark R. ^*,1^; Greenan, Garrett ^2^; Arora, Sumant ^3^; Murali, Arvind R. ^3^; Mayo, Marlyn J. ^1^. Ursodeoxycholic Acid Decreases Incidence of Primary Biliary Cholangitis and Biliary Complications After Liver Transplantation: A Meta‐Analysis. Liver Transplantation 27(6):p 866-875, June 2021. | DOI: 10.1002/lt.25935). For an adult with normal/average body weight, the doses of UDCA thus may be divided to three times a day.

  Query: Measurement of Specific Secondary Endpoints:

  "Bile Salt Export Pump levels (BSEP)": How will BSEP levels be measured?

 Perhaps I am mistaken, but BSEP is a membrane-bound transporter, so will

 only be measured with biopsies, as in you methods, will only be done in the

 suspicion of rejection. This is currently too vague.

 Reply: Thank you for your thoughtful feedback. You’re absolutely right—BSEP is a membrane‑bound canalicular transporter and, under normal conditions, is not released into the bloodstream in its intact form. In our study, however, we specifically sought to identify and quantify membrane‑derived fragments of BSEP that may be shed into the circulation during post‑transplant hepatocellular injury. To achieve this, we used a commercial sandwich ELISA kit validated for human serum and plasma. It is probable that the usefulness may not be as much as measuring the tissue BSEP levels, which will entail multiple liver biopsies in our patient. Our study results will hopefully give a better clue.

 Query: "Plasma bile acid levels":

  Clarification Needed: Will this be total plasma bile acids, or will specific bile

 acid species be fractionated and quantified (e.g., via LC-MS/MS)? The latter

 would be much more informative regarding FXR target engagement and

 changes in bile acid pool composition.

 Reply: Thank you for raising this important point. In our study, total plasma bile acid levels were quantified using commercially available ELISA kits. While we fully agree that fractionated bile acid profiling using LC-MS/MS would provide deeper insights into FXR target engagement and alterations in bile acid pool composition, this approach was not feasible in our current setting due to budgetary constraints and limited access to mass spectrometry facilities.

 Should we secure adequate funding in the future, we certainly plan to incorporate detailed bile acid species quantification via LC-MS/MS to enhance the mechanistic depth of our analysis.

  Definition of "Cellular Level" Assessment:

  The Rationale mentions "specific biochemical tests to assess cholestasis at the cellular

 Clarification Needed: Which specific tests (beyond standard LFTs and the

 listed secondary markers) are being referred to that assess cholestasis at a

 "cellular level"? This phrasing needs to be more precise or linked directly to

 specific planned assays (e.g., markers of oxidative stress, apoptosis, or specific

 transport protein expression if biopsies are analyzed beyond histology).

 Thank you very much for the comment. We have removed the usage of “cellular” from the context

 The text in the manuscript modified as below:

 This study aims to fill an essential gap by evaluating the efficacy of obeticholic acid compared to ursodeoxycholic Acid in managing post-liver transplant cholestasis. By conducting specific biochemical tests to assess cholestasis, this research will provide insights into the effectiveness of OCA relative to the current standard, UDCA.

 Query: Introduction and Conclusion Phrasing:

 Introduction: "Although its usefulness is questionable, many practitioners utilize

 ursodeoxycholic acid (UDCA)..." While there are debates, phrasing it as "questionable"

 without immediate specific referencing for the LT context might be too strong.

 Perhaps "its efficacy for preventing all forms of post-LT cholestasis or improving long

 term graft survival has limitations" or similar.

 Reply: Thank you very much for the comment. We have changed the sentence according to your suggestions.

 While many practitioners utilize UDCA as a hepatoprotective drug, its efficacy for preventing all forms of post liver transplant cholestasis or improving long-term graft server has limitations.

 Query:   Conclusion: "Obeticholic acid (OCA) is a potent farnesoid X receptor (FXR) agonist,

 with 100 times the efficacy of the current standard treatment, UDCA..." This is

 misleading. OCA is ~100 times more potent as an FXR agonist than chenodeoxycholic

 acid (a primary bile acid), not UDCA. UDCA is not a potent FXR agonist; it has different

 mechanisms of action (choleretic, cytoprotective, immunomodulatory). This

 statement needs correction for accuracy.

 Reply:Thank you very much for the comment. We have changed the sentence according to your suggestions.

 Sentence now reads as’ 

 Obeticholic acid (OCA) is a farnesoid X receptor (FXR) agonist, with significantly more potency than UDCA in modulating bile acid metabolism.

# Reviewer report: Reviewer response for version 1

**DOI:** 10.5256/f1000research.174896.r355921

**Published:** 2025-02-03

**Authors:** Palak J Trivedi

**Affiliations:** 1 University of Birmingham, Birmingham, UK

The rationale for the study is reasonable, but depth of secondary endpoints being captured is limited. Additionally, which adverse events will be captured, in what way, and how will they be recorded?

 The dose of UDCA is also lower than what would be considered standard practice.

 How will pruritus be captured? Highly relevant for OCA-treated patients.

 The pivotal phase III trial of obeticholic acid needs to be cited (Nevens et al. NEJM).

 As the authors may be aware, the EMA and FDA are in the process of revoking approval for obeticholic acid in Europe and the United States, respectively. Whilst such regulators do not necessarily have jurisdiction in India, how do the authors feel that these decisions will affect access to OCA?

Is the study design appropriate for the research question?

Yes

Is the rationale for, and objectives of, the study clearly described?

Yes

Are sufficient details of the methods provided to allow replication by others?

No

Are the datasets clearly presented in a useable and accessible format?

Partly

Reviewer Expertise:

Cholestasis, liver transplantation, clinical trials.

I confirm that I have read this submission and believe that I have an appropriate level of expertise to confirm that it is of an acceptable scientific standard, however I have significant reservations, as outlined above.

# Response

**Published:** 2025-06-23

**Authors:** Anila K N

**Affiliations:** GI Surgery, Not Applicable, Kochi, Kerala, India

**The rationale for the study is reasonable, but depth of secondary endpoints being captured is limited.**

 Thank you very much for the comments.

 Our secondary endpoints include all biochemical parameters (Liver function tests including Gamma Glutamyl transferase), post-transplant molecular markers fibroblast growth factor 19 (FGF-19), transforming growth factor β (TGF-β) level, Cytokeratin 18 level, Serum autotaxin level, Bile Salt Export Pump levels (BSEP), Plasma bile acid levels that assess biliary secretion. We did not include the pharmacodynamics and pharmacokinetics of obeticholic acid as it has already been done. We have also included health related quality of life assessment.

 We did not include any exploratory endpoints to formulate due to cost constraints.

**Which adverse events will be captured, in what way, and how will they be recorded?**

**&**

**How will pruritus be captured? Highly relevant for OCA-treated patients.**

 Thank You for the insightful query.

 Adverse events will be captured by direct interview of patients during the routine follow-up visits. The Naranjo algorithm will be used to establish causality. We will capture the pruritus by physical examination, vital signs and 5D pruritus questionnaire and will be documented systematically using structured Case Report Forms (CRFs) to ensure standardized data collection and analysis. The most common adverse event observed is pruritis.

 Pruritus associated with Obeticholic Acid (OCA) is known to be dose-dependent. However, in this study, we are using a lower dose of **5 mg once daily (OD)**, which is expected to minimize the incidence and severity of pruritus. Nonetheless, any pruritus reported by patients will be systematically assessed and documented in CRFs for consistency in data collection.

 The text will now read as:

 Adverse events are being captured by direct interview of patients during the routine follow-up visits. We are capturing the pruritus by physical examination, vital signs and 5D pruritus questionnaire and documented systematically using structured Case Report Forms (CRFs) to ensure standardized data collection and analysis. The Naranjo algorithm will be used to establish causality and then classify them as definite, probable, possible, or unlikely.

**The dose of UDCA is also lower than what would be considered standard practice?**

 Thank you for this comment.

 In fact, our recommended dose is 10 to 15 milligram per kilogram TID. Unfortunately, at one point the dose was mistakenly written as 10 to 15 mg/kg twice daily which is lower than the recommended dose, but shall be corrected.

 The text in the manuscript now reads as:

 It is usually administered for three months post-transplant at a dose of 10-15 mg/kg thrice  daily. In 2016, the FDA approved obeticholic acid (OCA), a strong selective Farnesoid X Receptor (FXR) agonist, to treat primary biliary cholangitis (PBC) in conjunction with UDCA. ^16^^, ^^17^ Research has demonstrated that over 12 months, OCA, either in combination with UDCA or on its own, dramatically lowers levels of total bilirubin and alkaline phosphatase. ^18^^, ^^19^ In individuals with advanced liver disease, the FDA has cautioned against administering OCA at doses of 10–50 mg. ^20^ Studies comparing OCA’s effects on cholestasis, rejection, regeneration, and cost-effectiveness to the current standard UDCA are being conducted to assess the role of OCA in the postoperative recovery of liver transplant patients. ^21^ Our proposed project is a clinical trial between OCA and the current standard of care, UDCA, in managing post-liver transplant cholestasis and improving graft survival.

**As the authors may be aware, the EMA and FDA are in the process of revoking approval for obeticholic acid in Europe and the United States, respectively. Whilst such regulators do not necessarily have jurisdiction in India, how do the authors feel that these decisions will affect access to OCA?**

 Thank you for the comment. We are aware of the recent FDA revoke for approval of obeticholic acid for further trials in steatohepatitis. However, we are not aware of any change in FDA approval for primary sclerosing cholangitis or primary biliary scleroses or similar cholestatic liver diseases.

 In any case, the caution against its use was for patients with advanced liver disease. In our scenario of post-liver transplant patients, that liver function is essentially normal and the dose that we use is much lower than the toxic doses that has been reported. We are using 5 milligram once a day. We hope that results of our trial may give further insight into the effectiveness and adverse effects of this drug in post-transplant population.
